# Association between the triglyceride glucose index and depression: a meta-analysis

**DOI:** 10.3389/fpsyt.2024.1390631

**Published:** 2024-06-20

**Authors:** Weitao Wan, Yi Yu

**Affiliations:** ^1^ Department of Psychiatry, Tianyou Hospital Affiliated to Wuhan University of Science and Technology, Wuhan, Hubei, China; ^2^ Department of Psychiatry, Wuchang Hospital Affiliated to Wuhan University of Science and Technology, Wuhan, Hubei, China

**Keywords:** depression, triglyceride glucose index, insulin resistance, risk factor, meta-analysis

## Abstract

**Background:**

Obesity and diabetes have been associated with depressive symptoms. The aim of this systematic review and meta-analysis was to evaluate the association between the triglyceride glucose index (TyG index) a novel indicator of insulin resistance (IR) and depression in the adult population.

**Methods:**

Relevant observational studies were acquired through comprehensive searches of the Medline, Web of Science, Embase, Wanfang, and China National Knowledge Internet databases. To account for heterogeneity, a random-effects model was employed to combine the findings. Additionally, multiple subgroup analyses were conducted to assess the impact of various study characteristics on the outcome.

**Results:**

The meta-analysis comprised eight datasets from six cross-sectional studies, encompassing a total of 28,973 adults. The pooled findings suggested that subjects with a high TyG index, compared to those with a low TyG index, were associated with a higher prevalence of depression (odds ratio [OR]: 1.41, 95% confidence interval (CI): 1.28–1.56, *p<*0.001; I^2 = ^19%). Sensitivity analyses, by omitting one dataset at a time, showed consistent results (OR: 1.39–1.45, *p*<0.05). Further subgroup analyses showed consistent results in participants aged <50 years old and in those aged ≥50 years old, in men and in women, in studies with different cutoff values for the TyG index, and in studies with different methods for the diagnosis of depression (for each subgroup difference, *p*>0.05).

**Conclusion:**

A high TyG index may be associated with a higher prevalence of depression in the adult population.

## Introduction

Depression is a severe and common affective disorder that adversely affects the quality of life of individuals (1, 2). Moreover, depression is associated with suicidal ideation, which may expose those affected to fatal events (3, 4). The pathogenesis of depression is complicated and far from clarified, but it is considered to involve a variety of genetic and environmental factors (5–7). Among them, metabolic disorders such as obesity (8) and type 2 diabetes (9) are both related to depression. The key component underlying obesity and type 2 diabetes is insulin resistance (IR) (10). Conventionally, the extent of IR can be accurately measured by the use of a hyperinsulinemic euglycemic clamp (11). However, this method is time-consuming, thus limiting its use in real-world clinical practice (11, 12). Recently, a novel indicator of IR based on the serum triglyceride (TG) and fasting plasma glucose (FPG) values has been proposed; this indicator is named the triglyceride glucose index (TyG index) and calculated on the basis of the equation: TyG index = ln [TG (mg/dL) × FPG (mg/dL)/2] (13, 14). It has been shown that the TyG index correlates well with IR as measured by a hyperinsulinemic euglycemic clamp (15–17). Moreover, compared to the conventional homeostasis model assessment of insulin resistance (HOMA-IR), the TyG index may more effectively predict the adverse events related to IR, such as early atherosclerosis (18), metabolic syndrome (19), and renal dysfunction (20). In view of the important association between IR and depression (21), it is necessary to evaluate the relationship between the TyG index and depression. However, previous studies evaluating the association between the TyG index and depression in the adult population have shown inconsistent results (22–27). Accordingly, a systematic review and meta-analysis was performed in this study to comprehensively address this issue.

## Methods

The present meta-analysis adhered to the guidelines outlined in the Preferred Reporting Items for Systematic Reviews and Meta-Analyses (PRISMA 2020) (28, 29) and the Cochrane Handbook for Systematic Reviews and Meta-analyses (30) throughout the process of study design, data collection, statistical analysis, and interpretation of results.

### Literature search

To identify studies relevant to the aim of this meta-analysis, we searched the Medline, Web of Science, Embase, Wanfang, and China National Knowledge Internet databases utilizing comprehensive search terms involving (1) “TyG index” OR “triglyceride-glucose index” OR “triglyceride and glucose index” OR “triglyceride glucose index” OR “triacylglycerol glucose index” OR “TyGI;” and (2) “depression” OR “depressive.” The search was restricted to human studies, specifically focusing on full-length articles published in peer-reviewed journals in English or Chinese. Additionally, the references of relevant original or review articles were manually examined to identify potentially pertinent studies. The literature encompassing the period from the establishment of the databases to December 6, 2023 was thoroughly screened.

### Inclusion and exclusion criteria

The eligibility criteria for the potentially included studies encompassed the following aspects, according to the PICOS criteria: The participants (P) included adults aged 18 years or older, without a diagnosis of a specific somatic disease. For the intervention/exposure (I), the TyG index was measured and the participants with the highest-category TyG index were considered as exposure. The TyG index was calculated as TyG index = ln [TG (mg/dL) × FPG (mg/dL)/2]. The cutoff for defining a high versus low TyG index was in accordance with the value used among the original studies. The participants with the lowest-category TyG index were considered as comparisons (C). To determine the outcomes (O), the incidence and/or prevalence of depression was compared between participants with the highest- versus the lowest-category TyG index. The study design (S) included observational studies, including case–control studies, cross-sectional studies, and cohort studies.

The exclusion criteria were as follows: (1) studies including children or adolescents; (2) studies including a patient population with a specific somatic disease; (3) studies that did not evaluate the TyG index or report the outcome of depression; or (4) preclinical studies, reviews, or editorials. If studies with an overlapping population were retrieved, the one with the largest sample size was included in the meta-analysis.

### Study quality evaluation and data extraction

The literature search, study identification, study quality evaluation, and data collection were carried out independently by two authors. In the event of any disagreement, consultation with the corresponding author was sought to resolve the matter. The Newcastle-Ottawa Scale (31) was employed to assess the quality of the studies included. This scale encompasses three dimensions, namely the selection of the study population, comparability between groups, and measurement of exposure. The score of NOS varied between 0–9, with a higher score indicating a better study quality. The NOS of 7–9 was defined as of good study quality. The data extracted from each study included various elements for subsequent analysis, such as the study information (including authors, year, country, and design), participant characteristics (including population characteristics, sample size, age, and sex), cutoff values for the TyG index analysis, methods for the diagnosis of depression, numbers of participants with depression, and variables adjusted when the association between the TyG index and depression was estimated.

### Statistical analysis

The relationship between the TyG index and depression in the adult population was summarized as the odds ratio (OR) and the corresponding 95% confidence intervals (CIs) comparing the prevalence of depression between participants with the highest- versus the lowest-category TyG index. ORs and standard errors were determined using 95% CIs or *p*-values, with a subsequent logarithmical transformation applied to stabilize and normalize the variance. Study heterogeneity was evaluated using the Cochrane Q test and I^2^ statistics, with an I^2^ value greater than 50% indicating significant heterogeneity (32). A random-effects model was employed to combine the results, taking into account the influence of heterogeneity (30). Sensitivity analyses, by omitting one study at a time, were conducted to further examine the findings. The study conducted predefined subgroup analyses to assess the impact of the study characteristics on the outcome. The medians of the continuous variables were utilized as thresholds to define the subgroups. To estimate publication bias in the meta-analysis, funnel plots were constructed and visually inspected for symmetry (33). Additionally, an Egger’s regression test was conducted (33). The statistical analysis was performed using RevMan (Version 5.1; Cochrane Collaboration, Oxford, UK) and Stata software (version 12.0; Stata Corporation, College Station, TX, USA). A two-sided *p*<0.05 indicates statistical significance.

## Results

### Study inclusion

**Figure 1:**
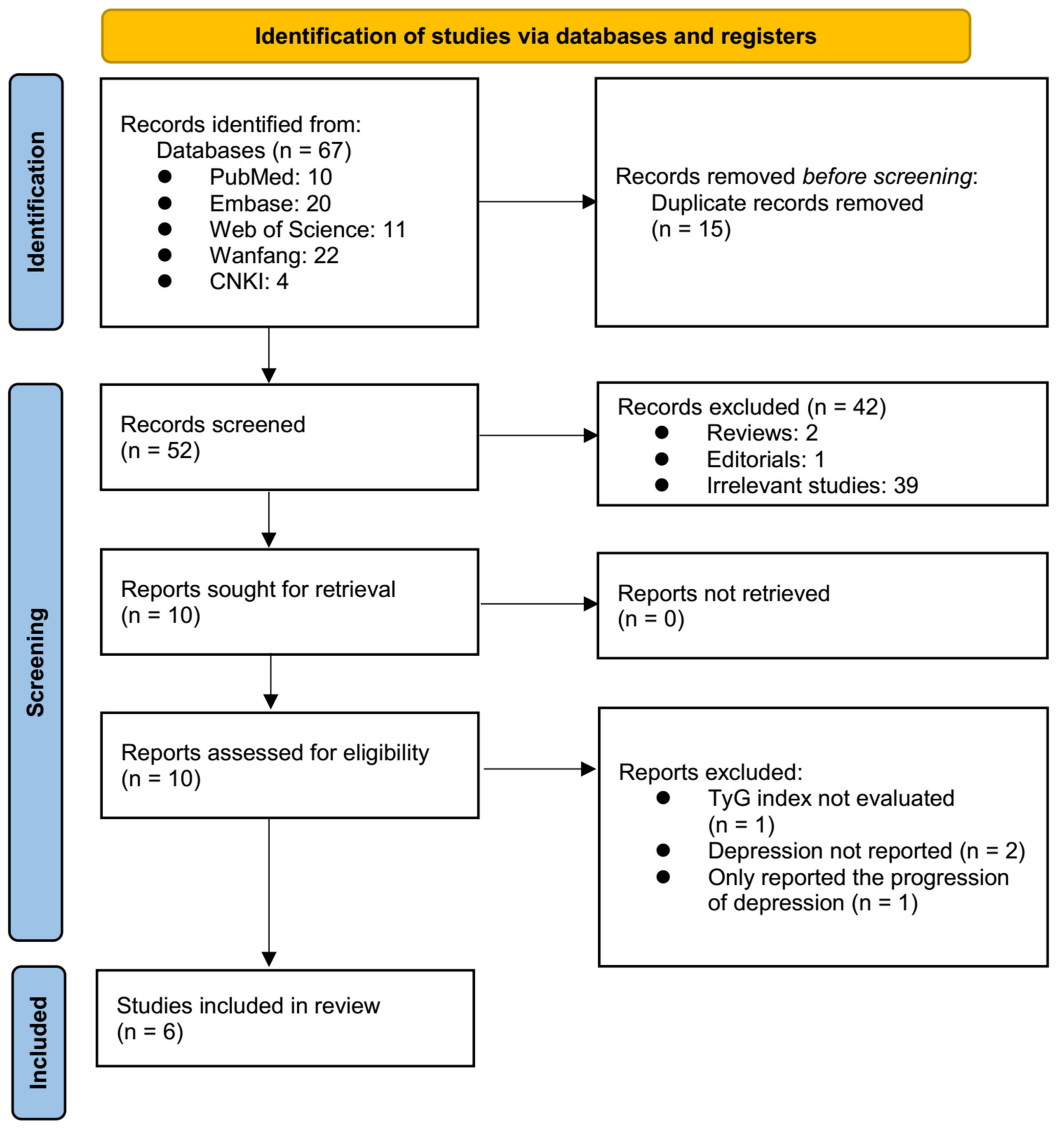
The flowchart depicts the process of database searching and study inclusion.

The process of study inclusion is presented in **Figure 1**. In brief, 67 potentially relevant records were obtained after comprehensively searching the five databases, and 15 of them were excluded due to duplication. Subsequently, screening of the titles and abstracts of the remaining records further excluded 42 studies, mostly because they were not related to the aim of the meta-analysis. Accordingly, the full texts of the ten resulting records were read by two independent authors, and four of them were further removed for the reasons listed in **Figure 1**. Finally, six observational studies were considered to be suitable for the subsequent quantitative analyses.

### Overview of study characteristics

**Table 1** presents the summarized characteristics of the included studies. Overall, six cross-sectional studies (22–27) were included in the meta-analysis. These studies were published between 2021 and 2023, and conducted in the United States, Korea, and China. Four studies included a community-derived adult population (22–24, 26), while the other two studies included adult participants who received a health examination (25, 27). The mean age of the participants was 44–59.1 years old, and the proportion of men was 17–54.2%. Medians (22, 26), tertiles (25, 27), and quartiles (23, 24) of the TyG index were selected as the cutoffs for the analyses of the TyG index in two studies each. Different methods were used to diagnose depression, including the Patient Health Questionnaire-9 (22, 23), the Center for Epidemiological Research Depression Scale 10 (24, 26), and the International Classification of Diseases 10 codes (25, 27). The assessment of patients that were diagnosed with depression was performed by trained medical personnel among all the included studies. Multivariate analyses were used to estimate the association between the TyG index and depression in all of the included studies, which adjusted potential confounding factors such as age, sex, body mass index, socioeconomic status, and comorbidities to a varying degree. The NOS of the included studies were summarized in **Table 2**. All of the included studies were of good study quality, with NOS between 7 and 9.

**Table 1:** Characteristics of the included studies.

Study	Location	Design	Population	No. of participants	Mean age (years)	Male (%)	TyG index analysis	Methods for depression diagnosis	Number of patients with depression	Adjusted variables
Shi 2021 (23)	USA	CS	Community population aged 20 years or older	13350	45.5	51.5	Q4:Q1	PHQ-9	1001	Age, race, sex, education, income, BMI, smoking, alcohol drinking, CHF, CAD, liver function, cancer, DM, and HDL-C
Lee 2021 (22)	Korea	CS	Community population aged 19 years or older	4688	46.8	54.2	Median	PHQ-9	422	Age, sex, marital status, personal income, smoking, DM, HTN, and CVD
Wang 2023 (26)	China	CS	Community population aged 45 years or older	8942	59.1	46.4	Median	CES-D-10	4345	Age, sex, education, marital status, live place, smoking, alcohol drinking, activities, exercises, and chronic diseases
Jiao 2023 (24)	China	CS	Community population aged 45 years or older	960	58.3	17	Q4:Q1	CES-D-10	NR	Age, sex, marital status, alcohol drinking, sleep duration, and chronic diseases
Jin 2023 (25)	China	CS	Participants receiving a health examination aged 18 years or older	646	44	42.7	T3:T1	ICD-10	321	Age, sex, BMI, marital status, family history of psychiatric disorders, education levels, hypertension, cardiovascular disease, stroke, cancer, and hypothyroidism
Zhang 2023 (27)	China	CS	Participants receiving a health examination aged 45 years or older	387	58.1	47.8	T3:T1	ICD-10	198	Age, sex, BMI, smoking, alcohol drinking, family history of depression, TC, HDL-C, and LDL-C

CS, cross-sectional; TyG index, triglyceride-glucose index; Q, quartile; T, tertile; PHQ-9, Patient Health Questionnaire-9; CES-D-10, the Center for Epidemiological Research Depression Scale 10; ICD-10, International Classification of Diseases 10; BMI, body mass index; CFH, congestive heart failure; CAD, coronary artery disease; CVD, cardiovascular disease; DM, diabetes mellitus; HTN, hypertension; TC, total cholesterol; HDL-C, high-density lipoprotein cholesterol; LDL-C, low-density lipoprotein cholesterol; NR, not reported;.

**Table 2:** Study quality evaluation via the Newcastle-Ottawa Scale.

Cross-sectional study	Adequate definition of cases	Representativeness of cases	Selection of controls	Definition of controls	Control for age and sex	Control for other confounders	Exposure ascertainment	Same methods for events ascertainment	Non-response rates	Total
Shi 2021 (23)	1	0	1	1	1	1	1	1	0	7
Lee 2021 (22)	1	0	1	1	1	1	1	1	1	8
Wang 2023 (26)	1	1	1	1	1	1	1	1	1	9
Jiao 2023 (24)	1	0	1	1	1	1	1	1	1	8
Jin 2023 (25)	1	0	1	1	1	1	1	1	1	8
Zhang 2023 (27)	1	0	1	1	1	1	1	1	1	8

### Results of the meta-analysis

**Figure 2:**
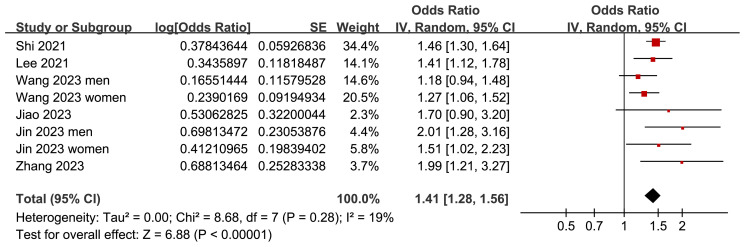
Forest plots for the meta-analysis of the association between the TyG index and depression in the adult population.

**Figure 3:**
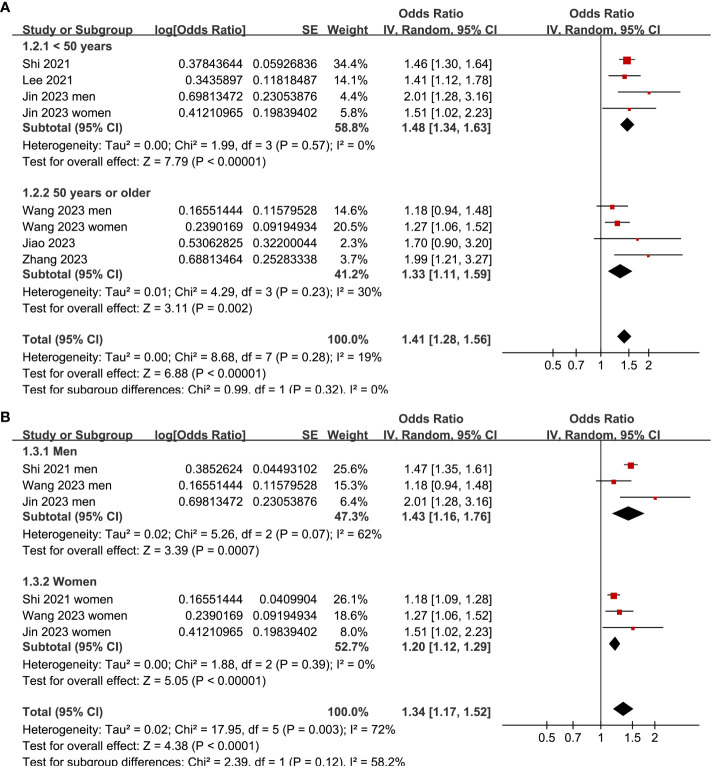
Forest plots for the subgroup analyses of the association between the TyG index and depression in the adult population. **(A)** Subgroup analysis according to the age of the participants. **(B)** Subgroup analysis according to the sex of the participants.

**Figure 4:**
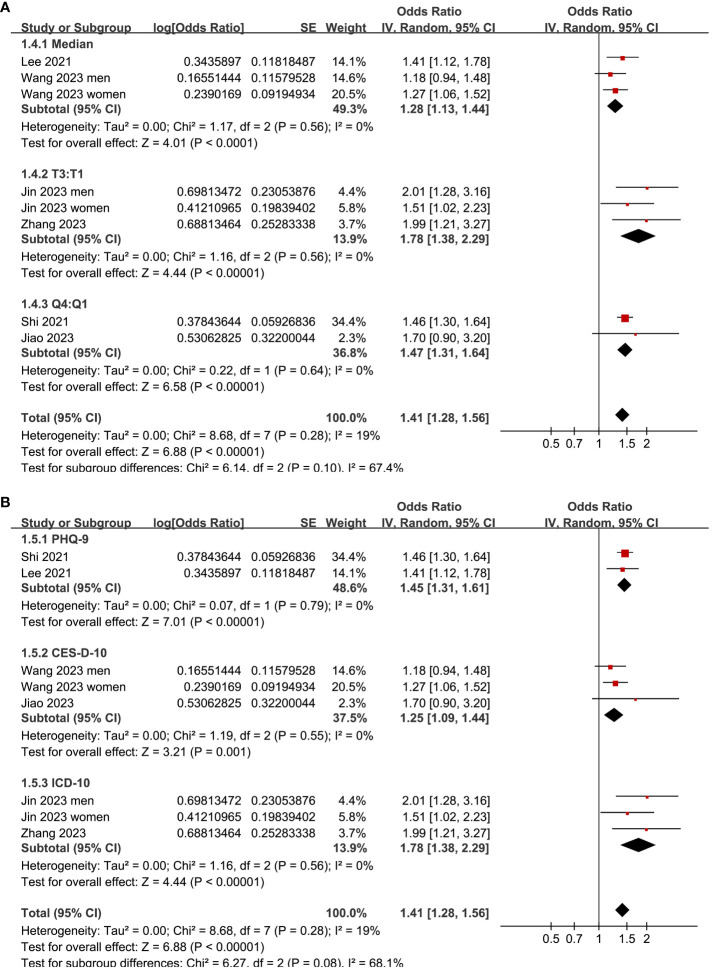
Forest plots for the subgroup analyses of the association between the TyG index and depression in the adult population. **(A)** Subgroup analysis according to the cutoffs of the TyG index. **(B)** Subgroup analysis according to the methods for the diagnosis of depression.

Two (25, 26) of the included studies reported the outcome according to the sex of the participants, and these datasets were included in the meta-analysis independently. Overall, the meta-analysis comprised eight datasets from six cross-sectional studies (22–27). The pooled findings suggested that the subjects with a high TyG index, compared to those with a low TyG index, were associated with a higher prevalence of depression (OR: 1.41, 95% CI: 1.28–1.56, *p<*0.001; **Figure 2**) with mild between-study heterogeneity (I^2^ = 19%). Sensitivity analyses, by omitting one dataset at a time, showed consistent results (OR: 1.39–1.45, *p*<0.05). Further subgroup analyses showed consistent results in participants aged <50 years old and in those aged ≥50 years old (*p*=0.32; **Figure 3A**), in men and in women (*p*=0.12; **Figure 3B**), in studies with different cutoff values for the TyG index (*p*=0.10; **Figure 4A**), and in studies with different methods for the diagnosis of depression (*p*=0.08; **Figure 4B**).

### Publication bias evaluation

**Figure 5:**
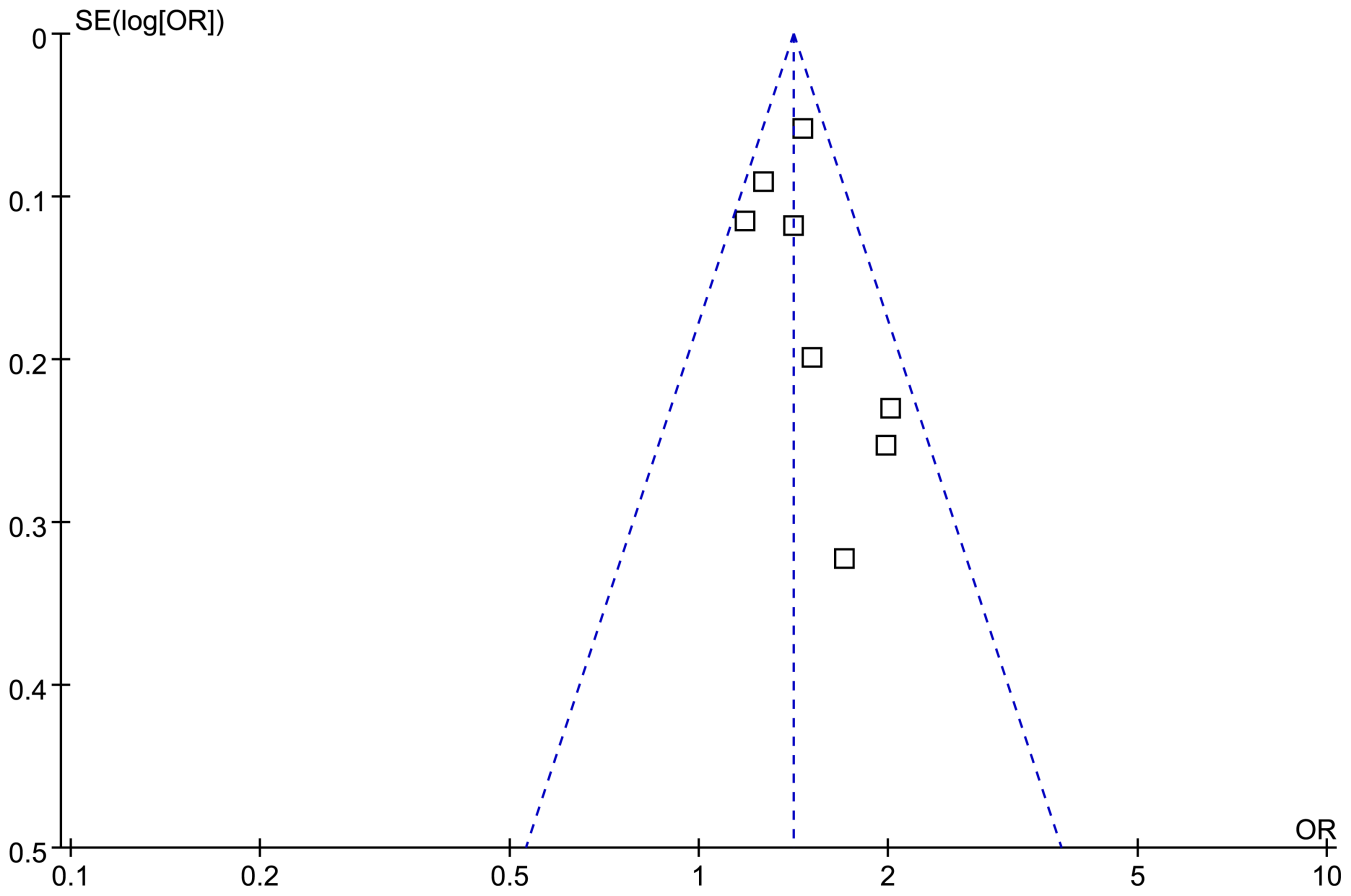
Funnel plots for the evaluation of the publication bias of the meta-analysis of the association between the TyG index and depression in the adult population.

The symmetrical nature of the funnel plots observed for the meta-analysis investigating the relationship between the TyG index and depression in the adult population suggests the absence of publication bias (**Figure 5**). This finding is further supported by the nonsignificant findings of Egger’s regression test (*p* = 0.25), which also suggested a low risk of publication bias.

## Discussion

This systematic review and meta-analysis synthesized the results of eight datasets from six cross-sectional studies and showed that in the adult population, a high TyG index was associated with a higher prevalence of depression. Furthermore, sensitivity analyses, which excluded one study at a time, consistently supported these results. Finally, subgroup analyses indicated that the correlation between the TyG index and the prevalence of depression in the adult population was not significantly affected by age, sex of the participants, cutoff values of the TyG index, or methods for the diagnosis of depression. In summary, the results of this meta-analysis collectively suggest that a high TyG index may be associated with a higher prevalence of depression in the adult population.

Based on our current understanding, this study represents the first systematic review and meta-analysis that comprehensively examines the potential relationship between the TyG index and depression in the adult population. Compared to the gold standard of hyperinsulinemic euglycemic clamp data or the commonly used HOMA-IR, the TyG index can be easily obtained via fasting TG and FPG measurements (34). Determining the TyG index is quick, cheap, and easy to implement in real-world clinical settings, which makes it a reliable and convenient indicator of IR (34). The results of our meta-analysis were consistent with previous findings, showing that both the TG and FPG levels are associated with depression. A previous meta-analysis including 11 case–control studies showed that an elevated TG level may be associated with first-episode major depressive disorder (35). Similarly, a recent study from the 2009–2018 National Health and Nutrition Examination Survey (NHANES) suggested that an elevated TG level may mediate the association between reduced physical activity and sedentary behavior in the development of depression (36). In addition, in a previous study of an urbanizing rural population in Bangladesh, it was shown that depression was significantly associated with a high FPG level (37). Similarly, in a subsequent study using the 2013–2014 NHANES data, it was demonstrated that a higher FPG level was associated with greater depressive symptoms among females, although the association was not significant in males (38). Collectively, the results of the current meta-analysis further confirmed the association between IR and depression.

Even though several mechanisms underlying the association between IR and depression have been elucidated, such as low-grade inflammation (39), changes of brain dopamine signaling (40), and cortisol dysregulation (41), the exact molecular pathways underlying this association remain to be clarified. For example, IR as indicated by a high TyG index, is linked to chronic inflammation and oxidative stress (39). Chronic low-grade inflammation, a hallmark of IR, can influence brain function by altering neurotransmitter metabolism, reducing neurogenesis, and impairing neuroplasticity, all of which have been implicated in the development of depressive symptoms (42). Additionally, IR is associated with increased levels of pro-inflammatory cytokines such as interleukin 6 and tumor necrosis factor α (43), which have been shown to affect mood and behavior by crossing the blood-brain barrier and impacting brain function. Furthermore, IR often correlates with disruptions in the hypothalamic-pituitary-adrenal (HPA) axis. These disruptions can lead to altered cortisol secretion patterns, which have been consistently linked to depression (44). Dysregulation of the HPA axis can exacerbate the body’s stress response, potentially contributing to depressive symptoms (45). Oxidative stress, another consequence of IR, can also lead to neuronal damage and affect brain regions implicated in mood regulation, such as the hippocampus and prefrontal cortex (46). The core signaling pathways underlying these proposed mechanisms remain to be determined.

Clinically, the findings of the meta-analysis underscore the significance of incorporating metabolic health evaluations into the management of depression. Screening for IR and related metabolic markers, including the TyG index, in individuals at risk for or experiencing depression could facilitate early intervention. Addressing IR through lifestyle interventions such as dietary changes and physical activity, or through pharmacological means when necessary, may improve both metabolic and mental health outcomes. This approach could lead to a more holistic treatment paradigm that considers both metabolic and psychological factors. Future research should aim to conduct longitudinal studies to better understand the temporal relationship between TyG index fluctuations and depressive symptoms. Investigating the detailed biological pathways, including the roles of inflammation, oxidative stress, and HPA axis dysregulation, could further elucidate the mechanisms driving the observed association. Additionally, exploring the impact of interventions targeting IR on depressive symptoms could provide valuable insights into potential therapeutic strategies. Such research could contribute to the development of more effective, multi-faceted treatment approaches for depression that address both metabolic and mental health components.

It is important to acknowledge the methodological rigor of this meta-analysis. Notably, a thorough search of five widely utilized electronic databases was conducted, resulting in the identification of six up-to-date cross-sectional studies that align with the objectives of this meta-analysis. All of the studies included in this analysis were published within the past three years, thus offering up-to-date insights into the role of the TyG index as a marker of depression in the general population. Furthermore, multivariate analyses were used for all of the included studies when the association between the TyG index and depression was estimated, therefore indicating that the association was independent of potential confounding factors, such as age, sex, or socioeconomic status of the participants. Finally, the robustness of the findings was further confirmed through various sensitivity and subgroup analyses. Taken together, these findings support the association between the TyG index and depression in the general population.

This study also has some limitations that must be addressed. One important issue is that the cutoff for the TyG index varied among the included studies. However, to the best of our knowledge, a universal cutoff for the TyG index for predicting IR remains to be established. Furthermore, all of the included studies were cross-sectional studies, and prospective studies should be performed to determine if a high TyG index is a risk factor for depression in the adult population. Moreover, we were unable to determine the potential influences of comorbidities and concurrent medications of the participants on the association between TyG index and depression because stratified data according to these characteristics of the participants were not reported among the included studies. However, participants with these conditions were rare among these studies because community population or general population were included. Additionally, our study solely encompassed observational studies, thus precluding the establishment of a causal relationship between IR and the development of depression. In fact, it is important to determine if there is a bidirectional relationship between a high TyG index and the prevalence of depression. In this regard, a recent study showed that major depressive disorder in adolescents appears to be a risk factor for development of dyslipidemia (47). Finally, although multivariate analyses were used, there might still be unadjusted factors that may confound the association between the TyG index and depression.

## Conclusion

In conclusion, the findings from this meta-analysis suggest that compared to those with a low TyG index, the adult population with a high TyG index may have a higher prevalence of depression. Although large-scale prospective studies are needed to validate the results, the findings of this meta-analysis support the potential association between a high TyG index and the prevalence of depression in general population.

## Data availability statement

The original contributions presented in the study are included in the article/supplementary material. Further inquiries can be directed to the corresponding authors.

## Author contributions

WW: Conceptualization, Data curation, Formal analysis, Investigation, Methodology, Resources, Software, Writing – original draft, Writing – review & editing. YY: Conceptualization, Data curation, Formal analysis, Investigation, Methodology, Project administration, Resources, Software, Supervision, Writing – original draft, Writing – review & editing.

## References

[1] Park LT Zarate CA Jr . Depression in the primary care setting. N Engl J Med. (2019) 380:559–68. doi: 10.1056/NEJMcp1712493 PMC6727965 30726688

[2] Malhi GS Mann JJ . Depression. Lancet. (2018) 392:2299–312. doi: 10.1016/S0140-6736(18)31948-2 30396512

[3] Grossberg A Rice T . Depression and suicidal behavior in adolescents. Med Clin North Am. (2023) 107:169–82. doi: 10.1016/j.mcna.2022.04.005 36402497

[4] Oliffe JL Rossnagel E Seidler ZE Kealy D Ogrodniczuk JS Rice SM . Men's depression and suicide. Curr Psychiatry Rep. (2019) 21:103. doi: 10.1007/s11920-019-1088-y 31522267

[5] Menard C Hodes GE Russo SJ . Pathogenesis of depression: Insights from human and rodent studies. Neuroscience. (2016) 321:138–62. doi: 10.1016/j.neuroscience.2015.05.053 PMC4664582 26037806

[6] Fox ME Lobo MK . The molecular and cellular mechanisms of depression: a focus on reward circuitry. Mol Psychiatry. (2019) 24:1798–815. doi: 10.1038/s41380-019-0415-3 PMC6785351 30967681

[7] Cai N Choi KW Fried EI . Reviewing the genetics of heterogeneity in depression: operationalizations, manifestations and etiologies. Hum Mol Genet. (2020) 29:R10–R8. doi: 10.1093/hmg/ddaa115 PMC7530517 32568380

[8] Jokela M Laakasuo M . Obesity as a causal risk factor for depression: Systematic review and meta-analysis of Mendelian Randomization studies and implications for population mental health. J Psychiatr Res. (2023) 163:86–92. doi: 10.1016/j.jpsychires.2023.05.034 37207436

[9] Genis-Mendoza AD Gonzalez-Castro TB Tovilla-Vidal G Juarez-Rojop IE Castillo-Avila RG Lopez-Narvaez ML . Increased levels of hbA1c in individuals with type 2 diabetes and depression: A meta-analysis of 34 studies with 68,398 participants. Biomedicines. (2022) 10. doi: 10.3390/biomedicines10081919 PMC9405837 36009468

[10] Hamer JA Testani D Mansur RB Lee Y Subramaniapillai M McIntyre RS . Brain insulin resistance: A treatment target for cognitive impairment and anhedonia in depression. Exp Neurol. (2019) 315:1–8. doi: 10.1016/j.expneurol.2019.01.016 30695707

[11] Gastaldelli A . Measuring and estimating insulin resistance in clinical and research settings. Obes (Silver Spring). (2022) 30:1549–63. doi: 10.1002/oby.23503 PMC9542105 35894085

[12] Park SE Park CY Sweeney G . Biomarkers of insulin sensitivity and insulin resistance: Past, present and future. Crit Rev Clin Lab Sci. (2015) 52:180–90. doi: 10.3109/10408363.2015.1023429 26042993

[13] Ramdas Nayak VK Satheesh P Shenoy MT Kalra S . Triglyceride Glucose (TyG) Index: A surrogate biomarker of insulin resistance. J Pak Med Assoc. (2022) 72:986–8. doi: 10.47391/JPMA.22-63 35713073

[14] Sanchez-Garcia A Rodriguez-Gutierrez R Mancillas-Adame L Gonzalez-Nava V Diaz Gonzalez-Colmenero A Solis RC . Diagnostic accuracy of the triglyceride and glucose index for insulin resistance: A systematic review. Int J Endocrinol. (2020) 2020:4678526. doi: 10.1155/2020/4678526 32256572 PMC7085845

[15] Mohd Nor NS Lee S Bacha F Tfayli H Arslanian S . Triglyceride glucose index as a surrogate measure of insulin sensitivity in obese adolescents with normoglycemia, prediabetes, and type 2 diabetes mellitus: comparison with the hyperinsulinemic-euglycemic clamp. Pediatr Diabetes. (2016) 17:458–65. doi: 10.1111/pedi.12303 26251318

[16] Guerrero-Romero F Simental-Mendia LE Gonzalez-Ortiz M Martinez-Abundis E Ramos-Zavala MG Hernandez-Gonzalez SO . The product of triglycerides and glucose, a simple measure of insulin sensitivity. Comparison with the euglycemic-hyperinsulinemic clamp. J Clin Endocrinol Metab. (2010) 95:3347–51. doi: 10.1210/jc.2010-0288 20484475

[17] Fiorentino TV Marini MA Succurro E Andreozzi F Sesti G . Relationships of surrogate indexes of insulin resistance with insulin sensitivity assessed by euglycemic hyperinsulinemic clamp and subclinical vascular damage. BMJ Open Diabetes Res Care. (2019) 7:e000911. doi: 10.1136/bmjdrc-2019-000911 PMC6861112 31798905

[18] Wang S Shi J Peng Y Fang Q Mu Q Gu W . Stronger association of triglyceride glucose index than the HOMA-IR with arterial stiffness in patients with type 2 diabetes: a real-world single-centre study. Cardiovasc Diabetol. (2021) 20:82. doi: 10.1186/s12933-021-01274-x 33888131 PMC8063289

[19] Son DH Lee HS Lee YJ Lee JH Han JH . Comparison of triglyceride-glucose index and HOMA-IR for predicting prevalence and incidence of metabolic syndrome. Nutr Metab Cardiovasc Dis. (2022) 32:596–604. doi: 10.1016/j.numecd.2021.11.017 35090800

[20] Gao W Wang J Chen Y Qiao H Qian X Xin Z . Discordance between the triglyceride glucose index and HOMA-IR in incident albuminuria: a cohort study from China. Lipids Health Dis. (2021) 20:176. doi: 10.1186/s12944-021-01602-w 34865646 PMC8647334

[21] Mehdi S Wani SUD Krishna KL Kinattingal N Roohi TF . A review on linking stress, depression, and insulin resistance via low-grade chronic inflammation. Biochem Biophys Rep. (2023) 36:101571. doi: 10.1016/j.bbrep.2023.101571 37965066 PMC10641573

[22] Lee JW Park SH . Association between depression and nonalcoholic fatty liver disease: Contributions of insulin resistance and inflammation. J Affect Disord. (2021) 278:259–63. doi: 10.1016/j.jad.2020.09.073 32977263

[23] Shi YY Zheng R Cai JJ Qian SZ . The association between triglyceride glucose index and depression: data from NHANES 2005–2018. BMC Psychiatry. (2021) 21. doi: 10.1186/s12888-021-03275-2 PMC8146990 34030657

[24] Jiao X Liu N Ma YW Zhang H Chen C Chen YC . Correlation between triglyceride-glucose index and depressive symptoms in middle-aged and elderly community population. J Clin Psychiatry. (2023) 33:272–5.

[25] Jin M Lv P Liang H Teng Z Gao C Zhang X . Association of triglyceride-glucose index with major depressive disorder: A cross-sectional study. Med (United States). (2023) 102:E34058. doi: 10.1097/md.0000000000034058 PMC10270554 37327285

[26] Wang Y Zhang X Li Y Gui J Mei Y Yang X . Predicting depressive symptom by cardiometabolic indicators in mid-aged and older adults in China: a population-based cross-sectional study. Front Psychiatry. (2023) 14:1153316. doi: 10.3389/fpsyt.2023.1153316 37351000 PMC10282944

[27] Zhang S Hou Z Fei D Zhang X Gao C Liu J . Associations between triglyceride glucose index and depression in middle-aged and elderly adults: A cross-sectional study. Med (United States). (2023) 102:E35530. doi: 10.1097/md.0000000000035530 PMC10615471 37904386

[28] Page MJ McKenzie JE Bossuyt PM Boutron I Hoffmann TC Mulrow CD . The PRISMA 2020 statement: an updated guideline for reporting systematic reviews. BMJ. (2021) 372:n71. doi: 10.1136/bmj.n71 33782057 PMC8005924

[29] Page MJ Moher D Bossuyt PM Boutron I Hoffmann TC Mulrow CD . PRISMA 2020 explanation and elaboration: updated guidance and exemplars for reporting systematic reviews. BMJ. (2021) 372:n160. doi: 10.1136/bmj.n160 33781993 PMC8005925

[30] Higgins J Thomas J Chandler J Cumpston M Li T Page M . Cochrane Handbook for Systematic Reviews of Interventions version 6.2. In: The cochrane collaboration. London, UK: Wiley Press (2021). Available at: www.training.cochrane.org/handbook.

[31] Wells GA Shea B O'Connell D Peterson J Welch V Losos M . The Newcastle-Ottawa Scale (NOS) for assessing the quality of nonrandomised studies in meta-analyses (2010). Available online at: http://www.ohri.ca/programs/clinical_epidemiology/oxford.asp.

[32] Higgins JP Thompson SG . Quantifying heterogeneity in a meta-analysis. Stat Med. (2002) 21:1539–58. doi: 10.1002/sim.1186 12111919

[33] Egger M Davey Smith G Schneider M Minder C . Bias in meta-analysis detected by a simple, graphical test. BMJ. (1997) 315:629–34. doi: 10.1136/bmj.315.7109.629 PMC2127453 9310563

[34] Tao LC Xu JN Wang TT Hua F Li JJ . Triglyceride-glucose index as a marker in cardiovascular diseases: landscape and limitations. Cardiovasc Diabetol. (2022) 21:68. doi: 10.1186/s12933-022-01511-x 35524263 PMC9078015

[35] Wei YG Cai DB Liu J Liu RX Wang SB Tang YQ . Cholesterol and triglyceride levels in first-episode patients with major depressive disorder: A meta-analysis of case-control studies. J Affect Disord. (2020) 266:465–72. doi: 10.1016/j.jad.2020.01.114 32056914

[36] Huang Y Xu P Fu X Ren Z Cheng J Lin Z . The effect of triglycerides in the associations between physical activity, sedentary behavior and depression: An interaction and mediation analysis. J Affect Disord. (2021) 295:1377–85. doi: 10.1016/j.jad.2021.09.005 34565593

[37] Natasha K Hussain A Azad Khan AK Bhowmik B . Prevalence of depression and glucose abnormality in an urbanizing rural population of Bangladesh. Diabetes Metab J. (2015) 39:218–29. doi: 10.4093/dmj.2015.39.3.218 PMC4696991 26706920

[38] Hoare E Dash SR Varsamis P Jennings GL Kingwell BA . Fasting plasma glucose, self-appraised diet quality and depressive symptoms: A US-representative cross-sectional study. Nutrients. (2017) 9. doi: 10.3390/nu9121330 PMC5748780 29215576

[39] Leonard BE Wegener G . Inflammation, insulin resistance and neuroprogression in depression. Acta Neuropsychiatr. (2020) 32:1–9. doi: 10.1017/neu.2019.17 31186075

[40] Gruber J Hanssen R Qubad M Bouzouina A Schack V Sochor H . Impact of insulin and insulin resistance on brain dopamine signalling and reward processing - An underexplored mechanism in the pathophysiology of depression? Neurosci Biobehav Rev. (2023) 149:105179. doi: 10.1016/j.neubiorev.2023.105179 37059404

[41] Joseph JJ Golden SH . Cortisol dysregulation: the bidirectional link between stress, depression, and type 2 diabetes mellitus. Ann N Y Acad Sci. (2017) 1391:20–34. doi: 10.1111/nyas.13217 27750377 PMC5334212

[42] Beurel E Toups M Nemeroff CB . The bidirectional relationship of depression and inflammation: double trouble. Neuron. (2020) 107:234–56. doi: 10.1016/j.neuron.2020.06.002 PMC7381373 32553197

[43] Hassamal S . Chronic stress, neuroinflammation, and depression: an overview of pathophysiological mechanisms and emerging anti-inflammatories. Front Psychiatry. (2023) 14:1130989. doi: 10.3389/fpsyt.2023.1130989 37252156 PMC10213648

[44] Hantsoo L Jagodnik KM Novick AM Baweja R di Scalea TL Ozerdem A . The role of the hypothalamic-pituitary-adrenal axis in depression across the female reproductive lifecycle: current knowledge and future directions. Front Endocrinol (Lausanne). (2023) 14:1295261. doi: 10.3389/fendo.2023.1295261 38149098 PMC10750128

[45] Leistner C Menke A . Hypothalamic-pituitary-adrenal axis and stress. Handb Clin Neurol. (2020) 175:55–64. doi: 10.1016/B978-0-444-64123-6.00004-7 33008543

[46] Ji N Lei M Chen Y Tian S Li C Zhang B . How oxidative stress induces depression? ASN Neuro. (2023) 15:17590914231181037. doi: 10.1177/17590914231181037 37331994 PMC10280786

[47] Khalfan AF Campisi SC Lo RF McCrindle BW Korczak DJ . The association between adolescent depression and dyslipidemia. J Affect Disord. (2023) 338:239–45. doi: 10.1016/j.jad.2023.06.017 37302507

